# Plastic Analysis with a Plasmonic Nano-Gold Sensor Coated with Plastic-Binding Peptides

**DOI:** 10.3390/jox14020040

**Published:** 2024-06-01

**Authors:** Francois Gagné, Maxime Gauthier, Chantale André

**Affiliations:** Environment and Climate Change Canada, Aquatic Contaminants Research Division, 105 McGill, Montréal, QC H2Y 2E7, Canada; maxime.gauthier@ec.gc.ca (M.G.); chantale.andre@ec.gc.ca (C.A.)

**Keywords:** plastic nanoparticles, peptide, nano-gold sensor, screening, freshwater mussels

## Abstract

Contamination with plastics of small dimensions (<1 µm) represents a health concern for many terrestrial and aquatic organisms. This study examined the use of plastic-binding peptides as a coating probe to detect various types of plastic using a plasmon nano-gold sensor. Plastic-binding peptides were selected for polyethylene (PE), polyethylene terephthalate (PET), polypropylene (PP), and polystyrene (PS) based on the reported literature. Using nAu with each of these peptides to test the target plastics revealed high signal, at 525/630 nm, suggesting that the target plastic limited HCl-induced nAu aggregation. Testing with other plastics revealed some lack of specificity but the signal was always lower than that of the target plastic. This suggests that these peptides, although reacting mainly with their target plastic, show partial reactivity with the other target plastics. By using a multiple regression model, the relative levels of a given plastic could be corrected by the presence of other plastics. This approach was tested in freshwater mussels caged for 3 months at sites suspected to release plastic materials: in rainfall overflow discharges, downstream a largely populated city, and in a municipal effluent dispersion plume. The data revealed that the digestive glands of the mussels contained higher levels of PP, PE, and PET plastic particles at the rainfall overflow and downstream city sites compared to the treated municipal effluent site. This corroborated earlier findings that wastewater treatment could remove nanoparticles, at least in part. A quick and inexpensive screening test for plastic nanoparticles in biological samples with plasmonic nAu-peptides is proposed.

## 1. Introduction

Plastics occur in many forms, including fibers, films, and particles, and are found in many terrestrial and aquatic environments, especially those near anthropogenic activities [1]. The size range of microplastic materials ranges from 1 µm to 5 mm and nanoplastics ranges from (1–1000 nm), although some classifications distinguish between nanoparticles (≤100 nm instead of 1000 nm). They also contaminate many food products, thereby exposing not only the biota but also the human population to their effects [2,3]. Despite some efforts to reduce plastic emissions by limiting single-use plastic products, they are still used as food wraps/containers for storage, and many other plastic-based materials are still used in our economy (thermoplastics surfaces, toys, various products). Moreover, since plastics degrade slowly, it is estimated that 710 million metric tons of plastic will found their way into the oceans by 2040 [4]. Large plastics could degrade into smaller and smaller particles down to the nanoscale due to natural weathering, sunlight, and erosion. 

Biofilms are formed on plastics during the degradation process, which is relatively lengthy depending on the polymer composition and its location [5]. Bacteria have been shown to have the capacity to adhere to plastic surfaces (anchor proteins/peptides) and to assist in degradation, forming a unique environment: the plastisphere. The fungi and bacteria found in these biofilms can degrade plastics such as polyurethanes [6,7]. These microorganisms produce a series of hydrolytic enzymes, such as esterase, lipase, proteases, and laccase, able to degrade polyesters. *Pseudomonas* sp. biofilms have been shown to have flexible metabolic activities involved in the oxidation of aromatic and aliphatic hydrocarbons found in plastics [8]. Peptides capable of interacting with plastics have been shown to contain tryptophan-X-X- tryptophan, phenylalanine-histidine-X-X-tryptophan, and tryptophan -X-X- tryptophan -X-X-tryptophan motifs, with X being any amino acids [9,10]. The amino acids Trp and Phe convey hydrophilic and cationic interactions from His and Gln, forming electro-attractive bonds on the negative charges found on plastic during weathering. These peptides have even been found to specifically bind specific plastic polymers, such as polystyrene (PS), polyethylene (PE), polyethylene terephthalate (PET), and polypropylene [11,12,13], hence the interest in using them in the development of detection systems for plastic materials, but the specificity of these peptides to other non-target plastics has not been determined. Conventional methods to detect plastics include pyrolysis gas chromatography, Fourier transform infrared/Raman spectroscopy, and transmission electron microscopy. These methods exhibit high selectivity and sensitivity but are expensive (instruments are costly), labor-intensive, and time-consuming, which limits their use on large scales for preliminary screening studies, hence the need for quick and rapid sensing technologies for various plastics.

Gold nanoparticles (nAU) are ideal substrates for optical sensing because of their high molar extinction coefficient, often visible by the naked eye, large surface area, and their optical properties related to size and distance distribution [14]. These nAu could bind typical biomolecules such as nucleic acids or proteins/peptides forming films (coatings) at the surface of nanoparticles. When these complexes are combined with test object, detection with high accuracy could be achieved using a simple visible spectrophotometer [15]. An interesting feature of nAu is that color changes are related to the intermolecular distance between nAu. Free nAu monomers display a pink-red (525 nm) to violet (>600 nm) color as the distance decreases proportionally between nAu (aggregation state). Aggregation could occur spontaneously or be induced by the addition of salts (NaCl) or acid (HCl). In the present study, nAu were coated with either PE-, PET-, PP-, or PS- binding peptides. The complex will bind to the corresponding target plastic fiber/particle, thereby protecting it against induced aggregation. In theory, plastic peptides binding to the various plastic polymers (PE, PET, PP, and PS) should be detectable by this technology. However, the cross-reactivity of these peptides with other plastics has not been determined. Indeed, the possibility exists that these peptides could cross-react with other plastic materials, since they are based on hydrophobic and cationic interactions.

The purpose of this study is, therefore, to develop a detection method based on a plastic-binding assay for PE, PET, PP, and PS nanoparticles using nAu–peptide complexes. The null hypothesis states that all tested peptides do not discriminate between the types of plastics. First, nAu will be coated with one of the above plastic-binding peptides and tested with the corresponding plastic polymer. Second, the specificity of each nAu–plastic peptide will be examined with non-target peptides in the attempt to determine any cross-reactivity. Thirdly, this newly developed assay will be tested in a case study involving mussels caged in three urban areas known to release plastic nanoparticles: two sites where street runoffs occur, in a municipal effluent plume, and downstream of a large city.

## 2. Methods

### 2.1. Preparation of nAu Peptides

Four peptides were synthesized by Canpeptides (Montreal, Québec, Canada) at a 10 mg scale based on reported sequences (see Table 1 for amino acid composition) and were destined for polyethylene (PE), polyethylene terephthalate (PET), polypropylene (PP), and polystyrene (PS) binding assays. They were dissolved in 50 mM NaCl containing 0.3 mM KH_2_PO_4_ and NaHCO_3_, pH 7.4 at 0.5 mg/mL, and stored at −20 °C. On the day of analysis, the peptides were diluted to 10 µg/mL in MilliQ water. Citrate-coated 10 nm diameter nAu were purchased from Nanocomposix (San Diego, CA, USA). The stock solution was 26 mg/mL in 2% citrate buffer as a stabilizing agent. The citrate nAu solution was centrifuged at 20,000× *g* for 10 min at 15 °C. The supernatant was carefully removed, and the pellet was resuspended in borosilicate tubes with the same volume of either 1 µg/mL PE- and PS-binding peptide or 0.25 µg/mL PET- and PP-binding peptide (Table 1). We used a lower concentration of PET- and PP-binding peptides to limit strong aggregation of the nAu suspension (appearance of dark violet coloration rather than the usual pink/red appearance). After standing at room temperature for 1–2 h, the suspension was centrifuged again (20,000× *g* for 10 min at 15 °C) to remove unbound peptides in the supernatant and the pellet was resuspended in MilliQ water. Preliminary experiments with fluorescently labeled peptides confirmed binding to nAu by the presence of fluorescence in the MilliQ washed nAu suspensions (between 5 and 20% of added fluorescence was retained on the nAu) [16].

### 2.2. Assay for PE, PET, PP, and PS Nanoplastics

PP and PE nanoparticles (0.1 µm diameter) were obtained from CD Bioparticles (New York, NY, USA). PS (0.1 µm diameter) and PET nanoparticles (1–3 µm diameter) were purchased from Polyscience (Niles, IL, USA) and nanoChemazone (Leduc, AB, Canada), respectively. The plastic samples were diluted at 10 mg/mL in MilliQ water. Previous experiments revealed that nanoparticle preparation in MilliQ water maintained the Zeta potential and prevented aggregation of the nanoparticles. For the assays, a standard curve between 1 and 100 µg/mL was constructed in MilliQ water at the day of the experiment. For the assay, 100 µL of nAu–peptide (either PE, PET, PP, or PS) was added to a clear microplate and increasing amounts of the corresponding target plastic were added and mixed for 5 min. After the addition of the aggregation agent (10 µL of 0.1 M HCl), plasmon resonance between 500 and 750 nm was measured using a microplate reader (Neo-2, Synergy, Biotek instrument, Santa Clara, CA, USA). The disaggregation ratio was calculated by the absorbance ratio at 525/620 nm. The specificities of each peptide against PE, PET, PP, and PS plastics were also determined. Each nAu–peptide was tested with the other non-target plastics (e.g., nAu-PS peptide tested with PE, PET, and PP and so forth) to determine any binding to the non-target plastics.

### 2.3. Application in Freshwater Mussels Caged in a Large Urban Area

The peptide-based assay was examined in freshwater mussels caged for 3 months (July–October, 2020) at 2 rainfall overflow sites (OVF1 and OVF2), one site located 15 km downstream from the city center of Montréal (Down City; QC, Canada), and a site downstream of the municipal effluent dispersion plume (Down Eff) discharged on the north shore of the Saint-Lawrence river as previously described [17]. The previous study revealed that micro- and nanoplastics are released in urban areas and that wastewater treatment plants can lower plastic micro- and nanoparticles in mussel tissues. Hence, the Down Eff site was considered the “reference” site. An operational blank with mussels maintained in the laboratory was also used and was below the detection limit of the methodology. A total of 30 mussels were placed in cylindrical nets and 3 nets were included at each site. The mussels were then placed at >1 m depth at the 4 sites described above from 15 July to 15 October. At the end of the exposure, the mussels were collected and allowed to stand for 12 h in clean aquarium water for purging of gut contents. The digestive glands were dissected on ice and homogenized in 100 mM NaCl containing 10 mM Hepes-NaOH, pH 7.4, 0.1 mM dithiothreitol, and 1 µg/mL protease inhibitor (aprotinin). Homogenization was performed with a polytron tissue grinder equipped with a steel probe for 30 s on ice. The homogenate was then extracted by adding one volume of saturated (5 M) NaCl and one volume of acetonitrile. This was left to stand for 30 min and mixed every 5–10 min [17]. The sample was then centrifuged at 500× *g* for 3 min to separate the phases. Preliminary experiments with fluorescently labeled nanoplastics revealed that plastics readily partitioned (>99%) in the acetonitrile phase. A 4x 10 µL volume was mixed with 140 µL of treated nAu toeach of the PE, PET, PP, and PS peptides in a clear microplate for 5 min, followed by the addition of 10 µL of 100 mM HCl. The samples were then analyzed by scanning spectrophotometry between 500 and 750 nm (2 nm increments, Neo-2 Biotech Instruments, Santa Clara, CA, USA). The disaggregation ratio was calculated at 525/620 nm. The blank consisted of 10 µL acetonitrile and standard solutions of each plastic particles were used (1–100 µg/mL). The data were expressed as relative levels of each plastics (µg/mg proteins). The total proteins in the digestive gland homogenate were determined using the Coomassie Brillant Blue assay using bovine serum albumin for calibration [18]. Other mussel physiological endpoints were included to seek out trends with bioavailable plastics in mussels as previously described [19]. These included the condition factor (mussel weight g/shell length cm), digestive gland index (DGI: g digestive gland/g tissues wet weight), gonado-somatic index (GSI; g gonad/g tissues wet weight), total lipids, and Nile Red solvatochromic shift (NR shift). Total lipids were determined using the microplate phosphovanilin methodology [20]. Calibration was achieved with canola oil and the data were expressed as µg lipids/mg proteins. The presence of plastic materials was determined using the solvatochromic properties of Nile Red, often used to stain plastics in various environmental samples [21]. The principle of this assay is based on the blue shift in the emission spectra of NR in tissues by non-polar materials such as plastics. This assay was designed as a semi-quantitative test for plastic-like materials based on the blue shift in the emission spectra of the dye in tissues. A 20 µL acetonitrile sample was mixed with 180 µM Nile Red (phosphate-buffered saline) and an emission scan was measured between 520 and 700 nm using an excitation wavelength of 490 nm (Neo-2, synergy, Biotech Instrument, Santa Clara, CA, USA). The first derivative spectra were calculated and the emission shift ratio was obtained (620 nm/650 nm emission). Standard solutions of PS nanoplastics were used for validation. The data were expressed as the shift ratio/mg proteins in the digestive gland. 

### 2.4. Data Analysis

All experiments for binding assays were run in triplicate (*n* = 3) and the data are expressed as the mean with standard deviation. For mussel exposure experiments, 4 individuals per site (*n* = 4) were analyzed. The data were analyzed using rank analysis of variance and critical differences between treatments were determined using the Conover–Iman test. The reference site was the treated municipal discharge site (with reported lower concentrations of nanoplastics). Correlation analysis was conducted using the Pearson moment procedure. To correct against the (lack of) specificity of some peptides, the residual values of each plastic were obtained from the levels of the other plastics: PS residual levels were obtained from the multiple regression of PS (dependent variable) with PE, PET, and PP (independent variable). The level of significance was set at α ≤ 0.05 and statistical analyses were conducted using the software Statsoft (version 13).

## 3. Results and Discussion

The plastic-binding peptides (see Table 1) used in the present study were synthesized to bind to PE, PET, PP, or PS nanoplastics. While PE an PP were aliphatic in nature, PS- contained aromatic styrene repeats, while the more polar PET-BP contained aromatic ester repeats. The peptide composition for each type of plastic is indicated based on the reported literature. The number of amino acids ranged between 7 and 13 amino acids. Hydrophobicity ranged between 43 and 67%. The peptide for PET and PE tended to aggregate nAu more strongly during the pre-incubation step, as evidenced by the appearance of a violet color. To limit this aggregation, 5 to 10 times less peptide was added during the peptide coating step for nAu. These peptides were generally more hydrophobic (54 and 67% for PET and PE, respectively), which could account for the stronger aggregation potential of nAu. The isoelectric point (IP) of the peptides for PE, PET, and PP was above nine, suggesting that these peptides were cationic at pH 7.4 and could interact with weathered plastics (containing R-OH and RCOOH ligands) and with Au^o^ at the surface of nAu. The PE peptide was the least polar, suggesting hydrophobic interactions at the surface of nAu, in keeping with the hydrophobic nature of PE (ethylene repeats). For PS-BP, the IP was closer to pH 7, which suggests more polar interactions between nAu and PS and the hydroxylated amino acids tyrosine and serine and perhaps some anionic interaction from the -COOH function of the terminal amino acid tyrosine.

**Figure 1:**
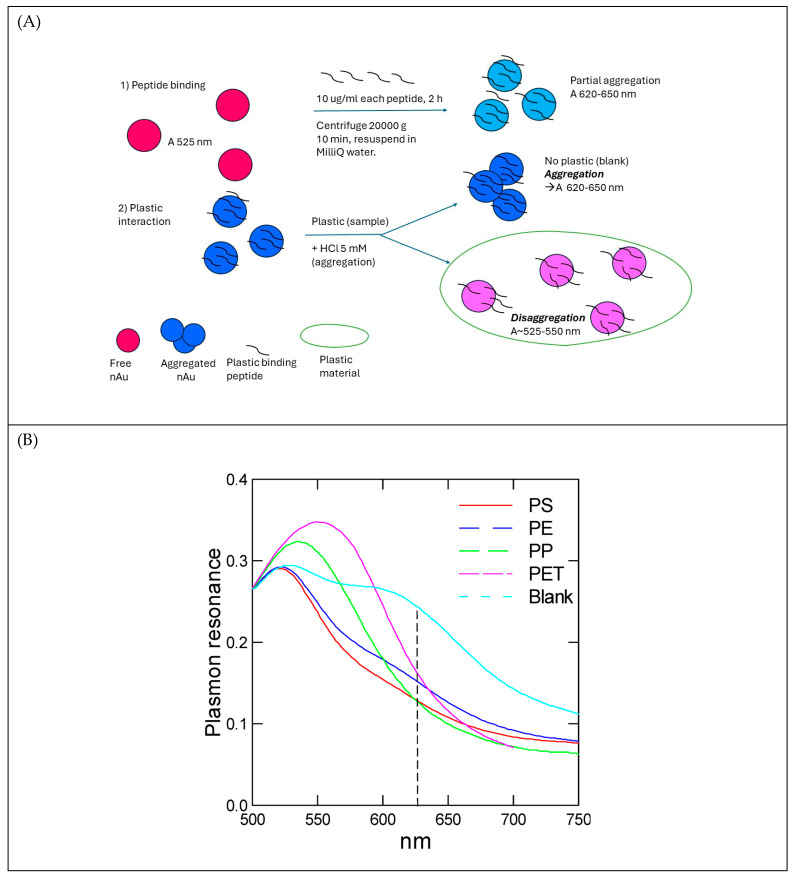
Plasmon resonance analysis of selected peptides. The principle of the plasmon assay using bacterial peptides is shown (**A**). Each peptide (PE, PET, PP, and PS) is incubated with nAu concentration to produce nAu-bound peptides. The nAu–peptides are then mixed with plastic samples (50 µg/mL), leading to reduced aggregation even after the addition of HCl. The plasmon resonance spectra in the absence of plastics (blank) and each of PE, PET, PP, and PS plastics with the corresponding nAu–peptide is shown (**B**). A blue shift in the resonance spectra is observed in the presence of each type of plastic with the corresponding peptide–nAu indicating disaggregation from plastics interaction with nAu-peptide.

The principle of this assay is based on the binding of a complex formed by nAu–peptide to plastic materials, which reduces the acid-induced aggregation (Figure 1A). Thus, it is expected that the aggregation of nAu induced by dilute acid dampens when nAu–peptide complexes interact with plastic materials, thereby causing an increased disaggregation ratio (525/630 nm). Peptides have previously been shown to bind to the surface of nAu through a combination of hydrophobic and ionic interactions [22]. Peptides with cysteine residues (R-SH), as with the PET peptide, can also form more stable (covalent) bonds to the surface of nAu by forming Au-S-peptide. The binding of peptides at the surface of nAu is relatively quick, requiring only few hours to form bonds. In some cases, thiol-containing agents could be incubated up to 12 h at room temperature, as with the mercaptoundecanoic acid ligand used as a general probe for nanoplastic detection in water samples [23]. The resonance spectra of each nAu–peptide with its corresponding plastic are shown in Figure 1B. The blanks consisting of the nAu–peptide only lead to two peaks around 530 and 600–630 nm, corresponding to a disaggregation ratio (530/620 nm) of circa 0.3/0.25 = 1.2. Although the maxima for aggregated nAu were closer to 600 nm, we selected 620 nm to reduce the spectral overlap with the various types of plastics. In the presence of plastics, the disaggregation ratio is increased up to 2.75 for PP, in agreement with the plastic preventing aggregation by HCl. It is also noticeable that the disaggregation of nAu is reduced for the PET peptide (the maximum is displaced from 525 to 550 nm), suggesting that nAu-PET peptide-PET were closer together compared to nAu-PS/PE/PP peptides when compared to the blank. The spectral shift (from 525 nm) is related to the relative distance between nAu particles following the plasmonic ruler phenomenon, which is used to estimate the relative distance between nAu [24]. The exact mechanism behind this observation is unclear but could be related to the lower amounts of cationic peptides remaining at the surface of nAu, offering less charge repulsion between nAu/PET and the peptide. Indeed, the nAu were treated with 0.25 µg/mL PET-BP, corresponding to about seven times less than the other peptides (based on molar values). The lower quantity of attached cationic PET peptides at the surface of nAu permits closer associations between nAu-PET and peptides by lowering charge repulsion from cationic peptides.

As plastic contamination in ecosystems often occurs as a complex mixture of several types of plastics, the specificity of each peptide was tested towards the four types of plastics used in this study (Table 2). Overall, the different peptides had the highest affinity with their respective theoretical plastic targets. However, they showed various affinities with the other plastics. The results showed that the nAu/PS peptide also responded to PE and PP, at 67 and 44% of the PS signal, respectively. The nAu/PET peptide showed reactivity with PS (28%), PE (38%), and PP (59%). nAu/PE-BP showed reactivity with PS (60%) and PP (34%). Finally, nAu/PP-BP showed reactivity with PS (50%) and PE (39%). If we consider 50% reactivity as a threshold, the PP peptide offered the best specificity compared to the other peptides. These results are indicative of cross-binding with other plastics. Such behaviors of peptides are to be considered carefully and used to generate relative measures of plastic charge, rather than absolute levels. A way to partially correct for the lack of specificity would be to subtract the signal (disaggregation ratio) of the target plastic with the other plastic signals (e.g., corrected nAu-PS signal = nAu-PS signal-(0.67* nAu-PET signal)-(0.44*nAu-PP signal)). A more robust method would be to extract the residual level of the target plastic with the other plastics using a multiple regression model: residual nAu-PS signal obtained from a multiple regression model of nAu-PS (as dependent variable) with PP, PE, and PET signals (as independent variables). Notwithstanding these correction strategies, only the relative levels of the target plastic could be obtained, not the absolute values. Confirmation by additional methodologies such as thermogravimetric Fourier transformation infrared spectroscopy (TG-IR) or pyrolysis gas chromatography–mass spectrometry (Py-GC-MS) would be required [25]. The peptides could be used together to coat the nAu sensor and provide a ‘total’ measurement of plastics (PS, PP, PE, and PET).

**Figure 2:**
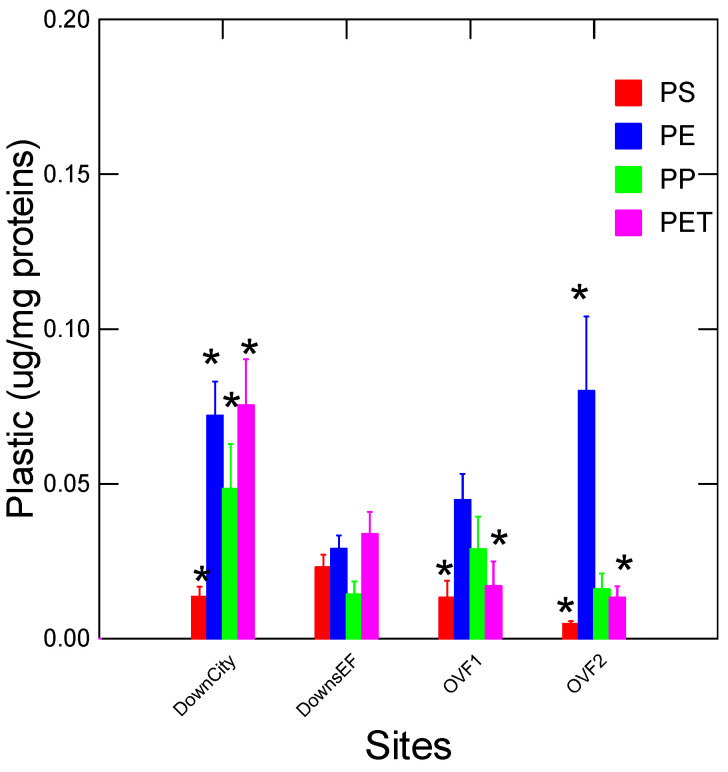
Relative levels of various types of plastics in freshwater mussels. The relative levels of plastics were obtained by correcting for interference using the residual method, as determined in Table 2. Data are expressed as the mean with standard error. The star symbol * indicates significance from the Down Effluent site.

As a case study, mussels were caged at two rainfall overflow sites (sources of tire wear plastics from street runoffs), downstream of a large city (an emitter of various plastic compounds), and downstream (8 km) of a municipal effluent dispersion plume (Figure 2). The relative levels of each plastic were obtained following the residual method described above. The data revealed that mussels placed at the Down Eff site had lower levels of PP, PE, and PET compared to the Down City site and some rainfall overflow sites. For PS, the levels in mussels at the Down Eff site were higher compared to the other sites. These results are supported by a study we previously published using flow cytometry and size exclusion chromatography using fluorescence detection to assess the total levels of nanoplastics in the digestive gland homogenates of mussels [17]. However, municipal effluents from three different cities were reported to emit circa 1 µg/L of nanoplastics [26]. Untreated effluents contained up to 30 µg/L nanoplastics and could be released during heavy rainfall at overflow sites. For the city of Montréal, this theoretically represents important amounts of plastics, since the station emits 2.5–7.5 × 10^9^ L/day (it is one of the highest effluent volume emitters in North America). Tires and asphalt are made from recycled plastics containing PE, PP, and PET, which are released by road wear in surface waters [27,28]. However, it is not known whether the plastic-binding peptides used here could bind to other plastics such as nylon, PVC, tire-related plastics (rubber), and polyesters. The removal rates of total nanoplastics were generally >90% for most PP, PP, PET, PVC, nylon, polymethylmethacrylate, and polycarbonate nanoplastics [26]. However, PS nanoplastics were sometimes less efficiently removed (43%) by primary and secondary treatment effluents; the effluent in which the mussels were caged was an advanced primary treatment. The mussels’ health status was determined by changes in condition factor (CF), digestive gland and gonad somatic indexes (DGI, GSI), total lipids in tissues, and the NR shift to detect low-polarity compounds in acetonitrile extracts (Table 3). The data revealed that the CF and lipid levels did not change across the sites, while the GSI and DGI were higher at the Down Eff sites compared to either the Down City or the overflow sites. The NR shift was higher at the OVF1 site and at the Down City site (containing higher levels of PE, PET, and PP plastics) compared to the Down Eff site. Furthermore, we tested the correlations between the variables (Table 4). In our study, we found positive correlations between plastics: PP and PE (*r* = 0.49) and PP and PET (*r* = 0.45). However, only the plastic PP showed correlations with other variables. PP was also positively correlated with the NR shift (*r* = 0.48) and lipids (*r* = 0.78). In addition, the NR shift was positively correlated with lipids (*r* = 0.49), and the DGI positively correlated with the GSI (*r* = 0.4). The associations between the variables PP, NR shift, and lipids are of particular interest as the PP peptide showed the be the most specific out of the four peptides. In other words, these correlations indicate that PP signals rise as concentrations of lipids increase, and this occurs conjointly with the NR shift. Although speculative, this correlation could be linked to the apolar nature of PP and the resulting interactions with lipids, which might enhance bioaccumulation in the digestive glands of mussels. An analysis of the covariance of the variable PP using lipids and DGI as covariates also revealed significant (*p* < 0.001) correlations between sites. This suggests that the observed increases in PP signals were not solely explained by lipid levels or changes in the DGI but by site-specific sources of plastics. 

In conclusion, this study revealed that plasmonic nano-gold sensors coated with plastic binding peptides can be used to track the relative levels of PE, PET, PP, and PS nanoparticles in biological tissue extracts. Although the plastic-specific peptides reacted the most with their corresponding target plastics, some cross-reaction with other plastics was found for the peptides used in the present study, which limits their direct application to environmental samples to measure absolute levels. A residual method based on multiple regression analysis is suggested to enhance the reliability of the assays revealed since each of the four tested peptides cross-reacted with non-target plastics. The relative levels of plastics could be obtained by extracting the residual levels of the plastics, i.e., the portion of plastic not related to the other interfering plastics. As a case study, mussels placed downstream from a large city, at rainfall overflow sites, and near a treated municipal effluent dispersion plume showed higher levels of total plastics at the large city and some overflow sites. Most plastics (PE, PET, and PP) were found at higher levels at the Down City and sometimes the OVF sites, with the exception of PS levels, which were higher in the treated municipal effluent plume compared to the other sites. A rapid and inexpensive peptide-based nAu sensor for the most abundant plastics PE, PET, PP and PS is proposed. Although the lack of specificity of some peptides towards non-target plastics could be mitigated by multiple regression analysis, the assay is limited to relative levels of plastics in biological samples. 

## Figures and Tables

**Table 1:** Plastic properties and plastic-binding peptides for nAu sensor.

Plastic	Structural Unit	Peptide sequence	# Amino Acids/ Hydrophobicity (%)/Isoelectric Point/Mass ^1^	Reference
Polystyrene (PS)	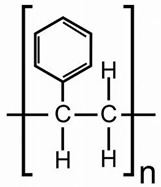	NH_2_-His-Trp-Gly-Met-Trp-Ser-Tyr-COOH	7 43% 6.74 966	[11]
Polyethylene terephthalate (PET)	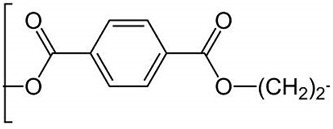	NH_2_-Cys-Trp-Phe Ala-Trp-Lys-Thr-His-Pro-Ile-Leu-Arg-Met-COOH	13 54% 9.51 1639	From [12]; Cys was added at the amino end in this study
Polyethylene (PE)	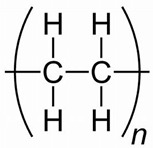	NH_2_-Leu-Pro-Pro-Trp-Lys-His-Lys-Thr-Ser-Gly-Val-Ala-COOH	12 67% 10 1320	[13]
Polypropylene (PP)	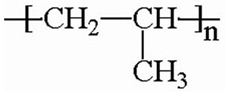	NH_2_- Met-Pro-Ala-Val-Met-Ser-Ser-Ala-Gln-Val-Pro-Arg-COOH	12 50% 9.5 1263	[13]

^1.^ Hydrophobicity index: (# of non-polar amino acids (Ala, Leu, Isoleu, Gly, Phe, Pro, Trp, Val)/total amino acids) × 100. Molecular mass in g/mole.

**Table 2:** Specificity of plastic-binding peptides.

nAu–Peptides	PS	PET	PE	PP
PS peptide	--	<1% ^1^	67%	44%
PET peptide	28%	--	38%	59%
PE peptide	60%	<1%	--	34%
PP peptide	50%	<1%	39%	--

Based on the following: (signal of other plastics/signal target plastic) × 100 for the same quantity of plastic (50 µg/mL). For example, the PS peptide was tested on PS, PET, PP and PE individually and the resulting signal at 525 nm/630 nm was measured and the target plastic signal is PS.

**Table 3:** General health status of caged mussels.

Sites	CF (Mussel g/cm)	DGI (g Digestive Gland/g Tissues)	GSI (g Gonad/g Tissues)	Lipids (ug Lipids/mg Proteins)	NR Shift ^1^
Down City	0.77± 0.03	0.033± 0.003	0.035± 0.003 *	1.32± 0.22	0.025± 0.002 *
Down Effluent	0.73± 0.03	0.037± 0.002	0.052± 0.004	1.37± 0.33	0.02± 0.001
OVF1	0.73± 0.025	0.031± 0.003 *	0.039± 0.004*	1.05± 0.14	0.03± 0.003 *
OVF2	0.77± 0.03	0.032± 0.002	0.039± 0.003 *	0.82± 0.21 *	0.018± 0.001

^1.^ Nile Red fluorescence shift ratio (emission 620/660 nm)/mg proteins in the digestive gland. The star symbol * indicates significance at *p* < 0.05.

**Table 4:** Correlation analysis.

	CF	DGI	GSI	NR_shift_	Lipids	PS	PE	PP	PET
DGI	−0.15	1							
GSI	−0.27	**0.4**	1						
NR_shift_	0.21	−0.14	−0.36	1					
Lipids	0.24	−0.07	−0.36	**0.49**	1				
PS	−0.15	0.28	0.12	0	0.25	1			
PE	0.001	0.27	0.12	0.04	0.2	−0.21	1		
PP	0.16	0.17	−0.28	**0.48**	**0.78**	0.18	**0.49**	1	
PET	−0.05	0.39	0	0.25	0.24	0.02	0.30	**0.45**	1

Significant correlations are highlighted in **bold** (*r* ≥ 0.4 at *p* ≤ 0.05).

## Data Availability

The data presented in this study are available on request from the corresponding author.
